# Determinants of cytoplasmic microtubule reorganization during ciliogenesis in *Chlamydomonas reinhardtii*

**DOI:** 10.1101/2023.04.07.536038

**Published:** 2023-05-03

**Authors:** Larissa L Dougherty, Prachee Avasthi

**Affiliations:** 1 Biochemistry and Cell Biology Department, Geisel School of Medicine at Dartmouth College, Hanover, New Hampshire

## Abstract

At the core of cilia are microtubules which are important for establishing length and assisting ciliary assembly and disassembly; however, another role for microtubule regulation on ciliogenesis lies outside of the cilium. The microtubule cytoskeleton is a highly dynamic structure which reorganizes rapidly to assist in cellular processes. These processes have been studied across various organisms with chemical as well as genetic perturbations. However, these have generated conflicting data in terms of the role of cytoplasmic microtubules and free tubulin dynamics during ciliogenesis. Here we look at the relationship between ciliogenesis and cytoplasmic microtubule dynamics in *Chlamydomonas reinhardtii* using chemical and mechanical perturbations. We find that not only can stabilized cytoplasmic microtubules allow for normal ciliary assembly, but high calcium concentrations and low pH-induced deciliation cause microtubules to depolymerize separately from ciliary shedding. In addition, we find that through mechanical shearing, cilia regenerate more quickly despite intact cytoplasmic microtubules. Our data suggests that cytoplasmic microtubules are not a sink for a limiting pool of cytoplasmic tubulin, reorganization that occurs following deciliation is a consequence rather than a requirement for ciliogenesis, and intact microtubules in the cytoplasm and the proximal cilium support more efficient ciliary assembly.

## INTRODUCTION

Ciliogenesis describes the formation of cilia which are microtubule extensions from the plasma membrane that serve as signaling and sometimes motile components of quiescent cells (Tucker et al., 1979). These largely microtubule-based structures are assembled and maintained through intraflagellar transport (IFT) where tubulin monomers among other components are taken to the tip of the cilium and back to the base by protein complexes (Craft et al., 2015; Kozminski et al., 1993). This process is influenced by not only the core proteins required for IFT, but also ciliary gene transcription, protein synthesis, trafficking, and complex pre-assembly occurring outside of the cilium. All of the required structures and components generate a highly regulated structure and signaling environment within cilia, and because of this, disruptions to any of these components can lead to various diseases (Reiter & Leroux, 2017). Therefore, it is important to understand how manipulation of cellular processes and components can influence ciliary assembly.

While cilia are themselves a microtubule superstructure, microtubules are also highly dynamic components elsewhere in the cell. They provide structure and polarity to the cell, serve as highways for molecular trafficking, and facilitate cell division among many other roles(de Forges et al., 2012; Parker et al., 2014). To serve these many roles, tubulin assembles and disassembles very rapidly through the process of dynamic instability which describes how tubulin can disassemble into monomers very quickly from GTP hydrolysis (Alushin et al., 2014; Mitchison & Kirschner, 1984).

Disruption of microtubule dynamics has been shown to directly impact the ability for cilia to assemble through chemical perturbations. For example, through the use of microtubule inhibitors such as colchicine or colcemid, cilia cannot assemble at all in Chinese hamster fibroblasts (Stubblefield & Brinkley, 1966) *Chlamydomonas* (Rosenbaum et al., 1969) and *Tetrahymena* (Rosenbaum & Carlson, 1969). Conversely, use of the microtubule stabilizer, taxol, has more recently been shown to inhibit acute ciliary elongation in *Chlamydomonas reinhardtii* (Wang et al., 2013). However, while microtubule destabilizing drugs were found to inhibit ciliary assembly, taxol was previously not found to have any effects in PtK1 cells on either assembly or disassembly of primary cilia (Jensen et al., 1987). In contrast, taxol can prevent actin stabilization and depolymerization-induced ciliary elongation through forskolin, jasplakinolide, and the PKA inhibitor CD. Consistently, the MT depolymerizing drug nocodazole was shown to increase ciliary length likely by freeing up tubulin for ciliogenesis in RPE cells whereas taxol itself shortened cilia (Sharma et al., 2011). Given these cell-type specific and chemical specific discrepancies describing the interplay between cytoplasmic microtubule dynamics and ciliary dynamics, it is important to continue investigating how these processes impact one another to better understand how ciliary dynamics are regulated.

Microtubule dynamics with respect to ciliary dynamics have predominantly been studied through chemical perturbations. Here we explore the requirement for microtubule dynamics in the ciliary model organism *Chlamydomonas reinhardtii* with both chemical perturbations and mechanical perturbations to better understand how microtubule dynamics can regulate ciliary assembly.

## RESULTS AND DISCUSSION

In our previous work, we found that a lower paclitaxel (PTX) concentration, 15 μM PTX, can allow for normal ciliary elongation following pH shock in *Chlamydomonas* (Dougherty et al., 2023). In addition, it is well known that higher concentrations of taxol, a paclitaxel equivalent, can induce different processes from lower concentrations, and lower concentrations of taxol can inhibit microtubule dynamics without altering tubulin polymer mass which occurs with higher concentrations (Derry et al., 1998; Jordan et al., 1993). Taxol can also differentially impact microtubule processivity depending on the organism and tubulin isoform (Chew & Cross, 2023).

**Figure 1.**
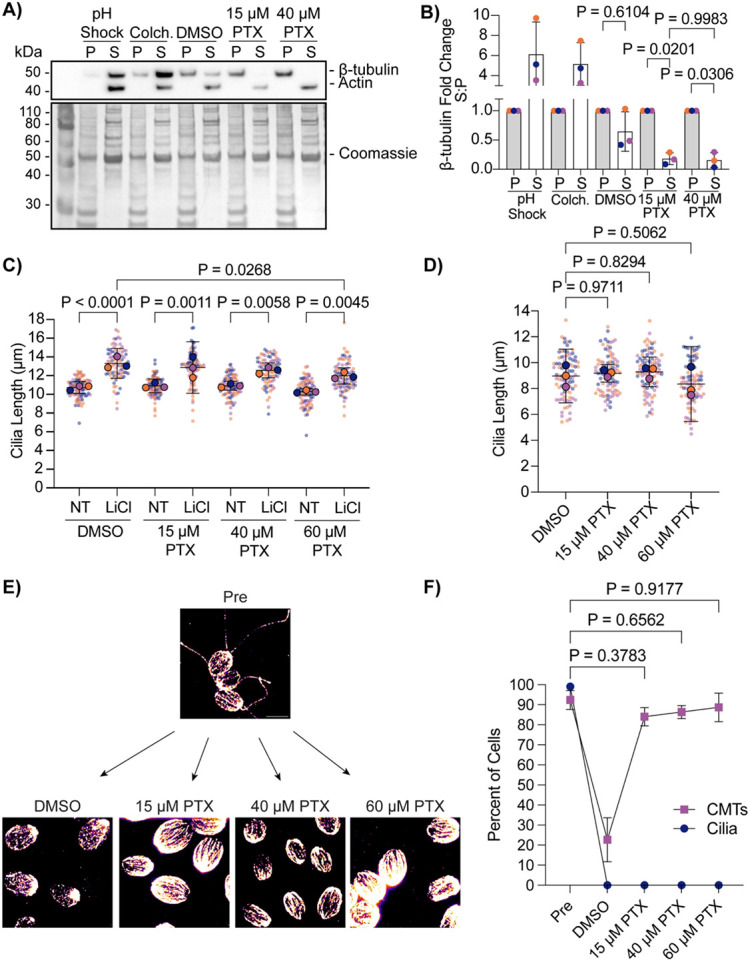
Paclitaxel-stabilized cytoplasmic microtubules permits normal ciliogenesis. **A)** Wild-type cells (CC-5325) were treated with acetic acid, 2 mg/mL colchicine, 1% DMSO, 15 μM paclitaxel (PTX), or 40 μM PTX. Protein was collected and probed for β-tubulin, actin (soluble protein control), or total protein with Coomassie on a PVDF membrane. **B)** Quantification of western blot band intensity in (**A**). Microtubule intensity was normalized to total protein and then the soluble tubulin (S) intensity was compared to the insoluble intensity (P). Significance was determined using a One-Way ANOVA with Šídák’s multiple comparisons test (N=3). Error bars are mean with standard deviation. For all tests, P<0.05 is considered significantly different. **C)** Wild-type cells (CC-1690) were grown in M1 media overnight and then treated with 1% DMSO or increasing concentrations of PTX with or without 25 mM LiCl for 30 min. Significance was determined with a One-Way ANOVA and Šídák’s multiple comparisons test (n=30, N=3). **D)** Wild-type cells (CC-5325) were grown in TAP media overnight, pretreated in increasing concentrations of PTX for 10 min, and then regenerated for 2 h after pH shock with acetic acid and PTX. Significance was determined with a One-Way ANOVA and Šídák’s multiple comparisons test (n=30, N=2). Error bars are mean with 95% confidence interval. **E)** Representative images of β-tubulin-stained cells at the 5 min timepoint post pH shock in (**D**). Scale bars are 5 μm. **F)** Quantification of the percent of cells with cilia and polymerized cytoplasmic microtubules (CMTs) at 5 min post pH shock in DMSO or PTX. Navy blue circles are cilia; purple squares are CMTs. Error bars are mean with standard deviation (n=100, N=3). Significance for CMTs is represented on the graph and determined using a One Way ANOVA with Šídák’s multiple comparisons test.

To test if taxol is indeed stabilizing microtubules at lower concentrations in *Chlamydomonas reinhardtii*, we isolated the soluble (“S”) and insoluble (“P”) β-tubulin fractions after treating cells with 0.5 M acetic acid to induce deciliation (“pH shock”) (Witman et al., 1972), 2mg/mL colchicine, 1% DMSO, 15 μM PTX, or 40 μM PTX (Figure 1A). Following PTX treatment, both 15 μM and 40 μM PTX decreased the presence of β-tubulin in the soluble fraction, confirming that both concentrations sufficiently stabilize microtubules in the cells (Figure 1A and 1B). To directly compare the effect of PTX on acute ciliogenesis extending beyond steady-state length, cells were treated with increasing concentrations of PTX in the presence or absence of 25 mM lithium chloride (LiCl) (Nakamura et al., 1987). Regardless of PTX concentration, cilia significantly elongated in the presence of LiCl in the CC-1690 wild-type strain in M1 liquid media (Figure 1C) previously used in (Wang et al., 2013). We also checked the ability for cilia to completely regenerate from zero length through pH shock in the presence of 15 μM PTX, 40 μM PTX, or 60 μM PTX compared to control cells in DMSO (Figure 1D). Similarly and consistently with our previous work (Dougherty et al., 2023), cilia regenerated normally in 15 μM PTX, though even at 60 μM PTX, ciliary length did not significantly differ from DMSO treated cells at 120 minutes (Figure 1D). To compare the ability for PTX to stabilize microtubules during pH shock, we pretreated cells for 10 minutes with PTX and then fixed and stained them for β-tubulin at 5 minutes post pH shock when microtubules are normally depolymerized (Figure 1E). We found that, even immediately following deciliation, microtubules are stabilized similarly to pre-deciliated cells (Figure 1F). Given that 1) there is already an appreciable pool of soluble tubulin at steady state without treatment, 2) decreasing this pool by inducing microtubule stabilization does not impact ciliogenesis, and 3) increasing this pool by depolymerizing microtubules through pH shock also does not impact ciliogenesis, our data suggest that tuning soluble tubulin access through cytoplasmic microtubule manipulation does not critically impact ciliogenesis.

**Figure 2.**
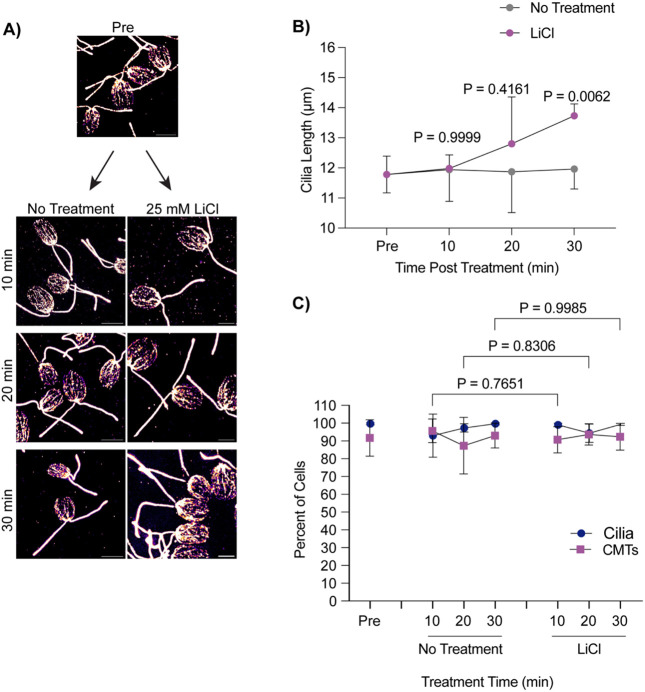
Lithium Chloride-induced ciliary elongation does not require microtubule depolymerization from steady state. **A)** Wild-type cells were treated with either 25 mM LiCl or no treatment for 30 minutes and then fixed and stained for β-tubulin. Scale bars are 5 μm. **B)** Quantification of cilia length for the experiment described in (**A**). Error bars are mean with 95% confidence interval (n=30, N=3). Significance compares cilia length at 30 min between treatments and was determined with a Two Way ANOVA with Šídák’s multiple comparisons test. **C**) Quantification of percent of cells with cilia versus polymerized microtubules for the experiment described in (**A**). Error bars are mean with standard deviation (n=100, N=3). Significance for CMTs is represented on the graph and determined using a One-Way ANOVA with Šídák’s multiple comparisons test.

While deciliation and regrowth of cilia from zero length is coincident with cytoplasmic microtubule depolymerization and stabilization of these microtubules does not affect cilium regrowth, we wondered whether acute cilium growth from steady state showed similar patterns of microtubule reorganization. Acute growth from multiple lengths may indicate whether cytoplasmic microtubule depolymerization is specific to cilium growth or deciliation. Using 25 mM LiCl to induce ciliary elongation, we checked the status of cytoplasmic microtubules across 30 minutes during treatment (Figure 2A). Throughout this time, cells were able to elongate ~2 μm as reported in Wang et al., 2013 (Figure 2B). However, upon checking cytoplasmic microtubule status throughout this process, we find that these microtubule arrays remained undisturbed in both untreated and LiCl treated cells (Figure 2C). This data suggests that microtubule reorganization is not necessary for ciliary assembly, though it is possible that finer microtubule reorganization could occur on a level that is not detectable with these methods.

**Figure 3.**
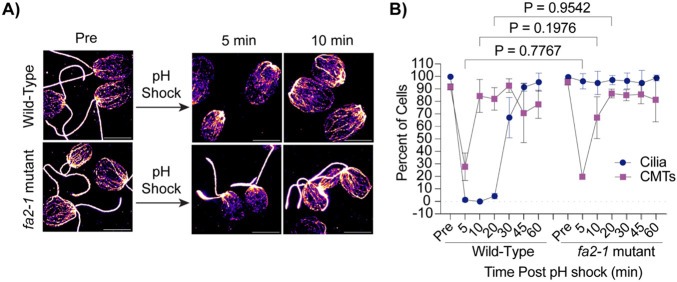
Cytoplasmic microtubule depolymerization occurs separately from ciliary shedding following pH shock. **A)** Wild-type or *fa2-1* mutants were pH shocked with acetic acid for 45 sec and then regrown in fresh tap for 60 min. Cells were fixed and stained for β-tubulin. Scale bars are 5 μm. **B)** Quantification of percent of cells with cilia or CMTs post pH shock. Error bars are mean with standard deviation (n=100, N=3). Significance was determined with a One-Way ANOVA and Šídák’s multiple comparisons test for comparisons between CMTs.

Ciliary shedding is also commonly induced through pH shocking cells (Witman et al., 1972). Microtubules depolymerize during pH shock-induced ciliary shedding (Wang et al., 2013) (Figure 1A and 1F) but do not depolymerize during LiCl induced ciliary elongation (Figure 2). Our data suggests that ciliary loss may trigger depolymerization rather than polymerization. To better understand if depolymerization is a result of physical loss of the cilium versus other factors coincident with cilium loss, we utilized a previously characterized *fa2-1* mutant encoding a mutant NIMA family kinase which cannot deciliated in the presence of calcium or acid (Mahjoub et al., 2002)(Figure 3A). Following pH shock, we found that while wild-type cells shed 100% of their cilia and depolymerized ~70% of their cytoplasmic microtubules (Figure 3B, **left**), the *fa2-1* mutants maintained cilia as expected, but surprisingly also depolymerized microtubules similar to wild-type (Figure 3B, **right**). Also interestingly, wild-type cells were able to reform their cytoplasmic microtubules within 10 minutes whereas ciliogenesis did not begin until between 20 and 30 minutes. This data shows that the presence of microtubules does not inhibit assembly given that microtubules are present at the time that ciliogenesis initiates, and cytoplasmic microtubule disassembly is a separate effect of pH shock from deciliation.

**Figure 4.**
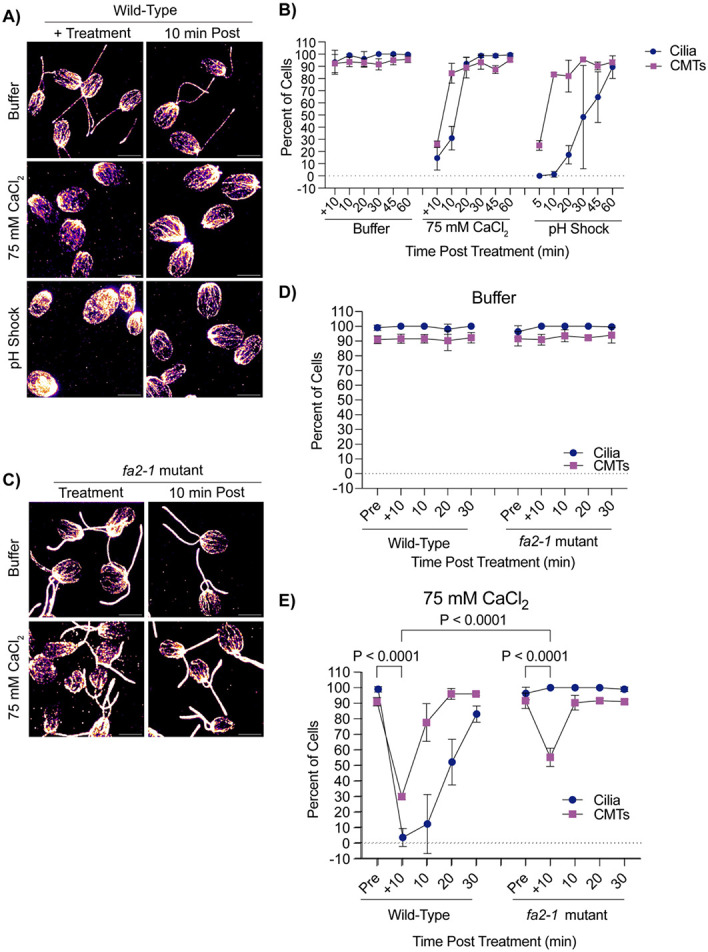
Cytoplasmic microtubule depolymerization occurs separately from ciliary shedding during high calcium. **A)** Wild-type cells were either pretreated for 10 minutes in 5 mM HEPES (“Buffer”), 10 minutes in 75 mM CaCl_2_, or 45 seconds in acetic acid, and then fixed and stained for β-tubulin. Scale bars are 5 μm. **B)** Quantification of cilia and CMTs from (**A**). Error bars are mean with standard deviation (n=100, N=3). **C)** Representative images of *fa2-1* mutants following treatment in buffer or 75 mM CaCl_2_. Cells were fixed and stained for β-tubulin. Scale bars are 5 μm. **D-E**) Quantification of wild-type (CC-5325) or *fa2-1* mutants in buffer (**C**) or 75 mM CaCl_2_ (**E**). Error bars are mean with standard deviation (n=100, N=3) for the experiment described in (**C**). Statistics compare CMTs and were determined using a One-Way ANOVA and Šídák’s multiple comparisons test.

It has previously been established that pH shock induces a calcium influx into the cell (L.M. Quarmby, 1996), and introducing the cell to higher concentrations of calcium can induce ciliary shedding (L. Quarmby & Hartzell, 1994). To further test what triggers microtubule reorganization coincident with deciliation we tested if calcium influx, known to occur upon pH shock was sufficient to induce cytoplasmic microtubule depolymerization. We introduced cells to 75 mM calcium chloride (“CaCl_2_”) in 5 mM HEPES (“Buffer”) for 10 minutes which is long enough to induce robust ciliary shedding. Following ciliary shedding, cilia regenerated similar to cilia exposed to pH shock (Figure 4A). We then measured the effect of calcium induced ciliary shedding on cytoplasmic microtubule stability. Upon ciliary shedding, we found that cytoplasmic microtubules also depolymerized quickly and then reformed similar to pH shock (Figure 4B). To determine if calcium has the same effect on ciliated cells, we repeated this experiment in *fa2-1* mutants (Figure 4C-E). Similar to wild-type cells, *fa2-1* mutants also exhibited depolymerized microtubules though they did not depolymerize to the same degree as wild-type (Figure 4E). It is possible that microtubule depolymerization could be occurring through similar mechanisms. It has previously been found that pH shock induces a calcium influx to activate ciliary severing (L. Quarmby & Hartzell, 1994). By flooding the extracellular matrix with high concentrations of calcium, it is possible that this is sufficient for calcium influx into the cell to activate the same pathways. In addition, it has previously been found that calcium destabilizes microtubule plus end growth (O’Brien et al., 1997). Calcium influx into the cell may be inducing multiple independent events within the cell.

**Figure 5.**
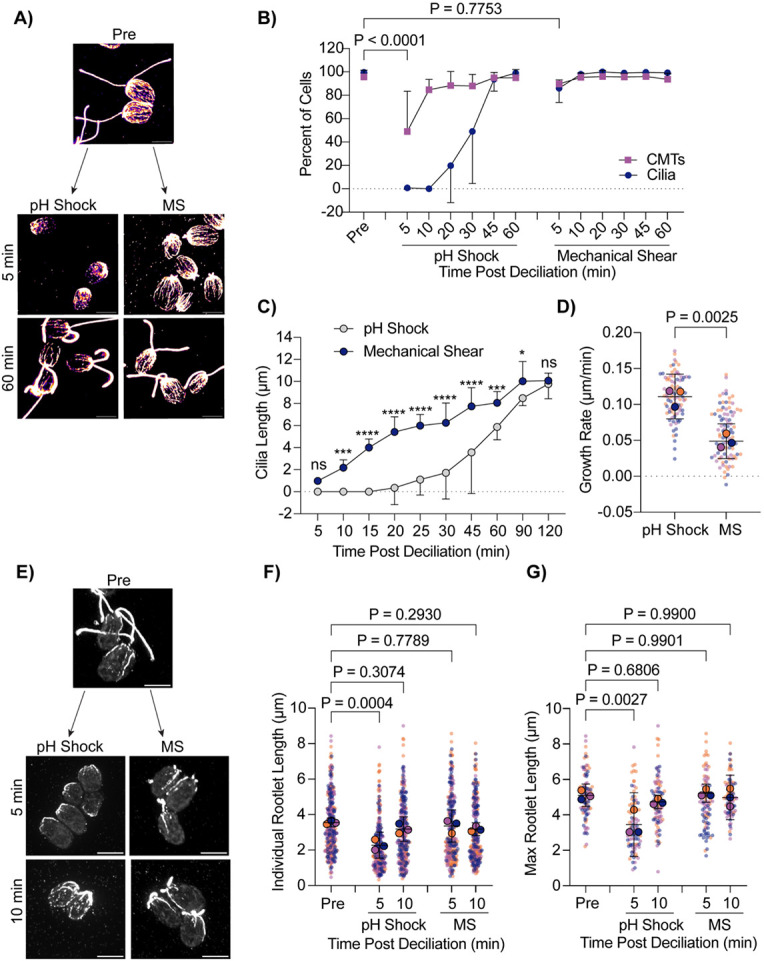
Cells maintain intact CMTs during mechanical shearing and regenerate cilia more quickly. **A)** Wild-type cells were either pH shocked for 45 s or placed in a cell homogenizer (“Mechanical Shear”, “MS”) for 3 minutes. Representative images are cells fixed and stained for β-tubulin. Scale bars are 5 μm. **B)** Quantification of percent of cells with cilia (navy circles) compared to polymerized microtubules (purple squares) between pH shock (left) and Mechanical Shear (“MS”, right). Error bars are mean with standard deviation. Statistics compare CMTs. Significance was determined using a One-Way ANOVA and Šídák’s multiple comparisons test (n=30, N=3). **C)** Quantification of cilia length over 2 hours. Error bars are mean with 95% confidence interval (n=30, N=3). Statistics were determined using a Two-Way ANOVA with Šídák’s multiple comparisons test (n=30, N=3). (ns - P>0.05, * P≤0.05, ** P≤0.01, *** P≤0.001, **** P≤0.0001). **D)** Quantification of the ciliary growth rate between pH shock and MS in (**C).** Error bars are mean with 95% interval. P values were measured using a t test (n=30, N=3). **E)** Wild-type cells were fixed and stained for acetylated α-tubulin following pH shock or MS. Scale bars are 5 μm. **F-G**) Quantification of individual **(F)** or max **(G)** acetylated microtubule rootlet lengths. Error bars are mean with 95% confidence interval. Statistics were determined using a One-Way ANOVA and Dunnett’s multiple comparisons test (n=30 cells, N=3).

Introducing different chemicals to cells to induce ciliary shedding induces differential effects on stability of cytoplasmic microtubules. We wanted to assess cytoplasmic microtubule stability without the use of chemicals. We introduced the cells to a cell homogenizer/bead beater for 3 minutes to excise cilia and then looked at cytoplasmic microtubules as cilia regenerated (Figure 5A). Interestingly, mechanical shearing did not induce robust cytoplasmic microtubule depolymerization (Figure 5B). Further, mechanically sheared cilia were able to regenerate back to full length much faster (in 60 minutes) whereas cilia shed via pH shock were only able to regenerate to half-length in this time (Figure 5C). Looking more closely at regeneration in these processes, we found that mechanically sheared cilia, which maintain a ~1 μm ciliary track immediately following ciliary excision, can immediately begin steadily regenerating overtime (Figure 5C) at a slower rate (Figure 5D). In contrast, pH shocked cells have delayed ciliogenesis, but after this process is started, cells can more rapidly regenerate cilia (Figure 5B-C). To confirm that this was not a consequence of cytoplasmic microtubule reestablishment, we compared ciliogenesis to cytoplasmic microtubule organization. Though pH shocked cells reestablished microtubules within 10-20 minutes, ciliogenesis did not continue until 30-45 minutes post deciliation unlike mechanically sheared cells which maintained both cytoplasmic microtubule organization and cilia throughout (Figure 5B-C). These data show that in the case where cilium growth happens faster, depolymerization does not occur. Our data may suggest that polymerized cytoplasmic microtubules facilitate more rapid ciliogenesis. Alternatively, the existence of cilium initial segments may promote more rapid assembly completely independent of cytoplasmic microtubules state that is dependent instead upon calcium influx (Figure 4) that may not be triggered upon mechanical shearing. It could be that initiation of ciliary assembly is rate limiting.

The delay in ciliary shedding due to pH shock may differentially affect microtubules based on modifications that add stability. To test this, we also compared the presence of acetylated α-tubulin in pH shocked cells versus mechanically sheared cells. Deacetylation of α-tubulin has been found to work in concert with ciliary disassembly during mitosis, and acetylation can act as a signal for downstream events such as cell differentiation in adipocytes (Forcioli-Conti et al., 2016). In addition to the ciliary axoneme being heavily comprised of acetylated tubulin, *Chlamydomonas* has 4 rootlet microtubules present at the ciliary base which extend into the cell (Boyd et al., 2011; Dutcher & O’Toole, 2016). Acetylation recruits further modifications to tubulin that allow for motility and ciliary length maintenance in *Chlamydomonas* as well as human cells (Tripathi et al., 2021). It is possible that pH shock could be inducing upstream pathways that inhibit tubulin acetylation must be maintained for ciliogenesis. Comparing pH shock to mechanical shearing, we found that during pH shock, acetylated microtubules were significantly shorter both overall (Figure 5E and F) and when comparing the longest rootlet microtubule per cell (Figure 5E and G). These data show that pH shock may affect both stabilized and non-stabilized microtubules or affect the degree of acetylation of the stabilized population.

Delayed ciliogenesis could indicate that there is a recruitment defect for proteins necessary for ciliogenesis. Using a previously tagged mScarlet-IFT54 anterograde IFT subunit and sfGFP tagged retrograde IFT140 subunit generated and validated by (Wingfield et al., 2021), we compared fluorescence of these complexes at the ciliary base during pH shock and mechanical shear (Supplemental Figure 1A and 1C). We found that during pH shock, there is a significant and more robust increase in both anterograde (Supplemental Figure 1A and 1B) and retrograde (Supplemental Figure 1C and 1D) trafficking complexes during pH shock as compared to mechanical shearing which remains increased at 10 minutes when cytoplasmic microtubules have reformed (Figure 3B). Together, this data suggests that pH shock may cause a delay in ciliogenesis due to altered IFT recruitment at the base of cilia which is required for participation in ciliogenesis. While recruitment is still induced during mechanical shear, there is a ~1 μm track of cilia present for IFT to escape from the basal body area of the cilium which could explain the reduced basal body accumulation due to some IFT components departing for the nascent cilium. This data also may suggest that presence of established microtubules can actually enhance ciliogenesis. It is possible that either through having established acetylated microtubules or cytoplasmic microtubules without acetylation, molecular highways do not have to be rebuilt for proteins from the ciliary precursor pool to be mobilized to the ciliary base.

Throughout this work, we identify the relationship between ciliogenesis and cytoplasmic microtubule polymerization which we tease apart by holding either of these processes constant while varying possible determinants. We find that while microtubule depolymerization does occur during deciliation with some techniques to induce deciliation, these dynamics are not necessarily required for ciliary assembly and instead may inhibit ciliary assembly as we see a reestablishment in acetylated microtubule length before ciliogenesis can begin (Figure 5 E-G) and a need for IFT recruitment to the ciliary base (Supplemental Figure 1). These findings are summarized in Supplemental Figure 2. Our data are consistent with a model in which events happening during deciliation have effects on associated cytoplasmic microtubule processes, but these are not required for ciliogenesis. These data shed light on two important points: 1) tubulin does not necessarily need to be freed up from the cell in order to allow acute ciliary assembly to occur; and 2) soluble cytoplasmic tubulin are neither required nor block the mobilization of the intracellular pool of ciliary protein precursors which we see through stimulated IFT recruitment both during pH shock and mechanical shearing, though this response is less robust during mechanical shearing.

Effects of pH shock and calcium on live microtubule dynamics alone have been investigated (Liu et al., 2017), however the stability of existing microtubules has not previously been fully explored. Liu et al., 2017 found that, by using a Neon-Green tagged EB1 protein, microtubule growth was frozen in *Chlamydomonas* by decreasing pH and increasing calcium concentration. They also noted that the different *Chlamydomonas* tubulin isoforms have different isoelectric points (α-tubulin = 5.01, β-tubulin = 4.82, and EB1 = 5.7), so dropping the pH below these values which allows for sufficient calcium influx to induce ciliary shedding is also sufficient to separately induce microtubule depolymerization, along with the ability for calcium alone to inhibit microtubule dynamics. However, despite the effect of pH on microtubule stability shown in this paper, there is also a considerable lag between the time that microtubules are reestablished the timing of initial ciliogenesis (Figure 3B and 5B) which raises the possibility that other signaling pathways that influence ciliogenesis are inhibited during this process. For example, it was previously found that pH shock activates the microtubule depolymerizing kinesin CrKin13 which has also been implicated in ciliogenesis (Wang et al., 2013). Future work needs to be done to better understand how different methods of deciliation can ultimately impact cellular processes that directly regulate ciliogenesis.

## MATERIALS AND METHODS

### Strains and Maintenance

The wild-type strains (CC-5325 and CC-1690), *fa2-1* mutant (CC-3751), and IFT strain (ift140-1::FT140-sfGFP ift54-2::mS-IFT54 mt; CC-5862) were acquired from the *Chlamydomonas* Resource Center. Cells were maintained on 1.5% Tris Acetate Phosphate (TAP) plates under constant light. For experiments, CC-1690 cells were inoculated into liquid M1 media, and CC-5325 cells, *fa2-1* mutant cells, and IFT cells were inoculated in tap and grown overnight under constant light and agitation.

### Ciliary Length Experiments

#### Steady State Ciliary Length Experiments:

overnight cultures were resuspended in fresh TAP or M1 (CC-1690) the next morning. Cells were treated with either DMSO, taxol (PTX), colchicine, or LiCl, fixed in equal amounts of 2% glutaraldehyde, and then imaged on a Zeiss Axioscope 5 DIC with 40x magnification and Zeiss Zen 3.1 software.

#### PH Shock Induced Ciliary Shedding and Regeneration:

overnight cultures were resuspended in fresh TAP and pretreated or not with PTX 10 minutes prior to deciliation. To induce deciliation, 0.5 M acetic acid was added to the cell suspension to bring the pH down to 4.5 for 45 sec, and then brought back up to 7.0 with 0.5 M potassium hydroxide. Cells were then spun down at 600xg for 1 minute and resuspended in new tap with or without PTX.

#### Calcium Chloride-Induced Ciliary Shedding and Regeneration:

overnight cultures resuspended in fresh tap were spun down and resuspended in either 5 mM HEPES or 75 mM calcium chloride in 5 mM HEPES for 10 minutes. Then cells were resuspended in fresh media and allowed to regenerate cilia.

#### Mechanical Shear-Induced Ciliary Shedding and Regeneration:

Overnight cultures resuspended in fresh tap were placed in a cell homogenizer (Analog Disruptor Genie) for 3 minutes and then resuspended in fresh TAP to regenerate.

### Immunofluorescence and Quantification

#### β-Tubulin:

Staining was performed as described in (Wang et al., 2013). Briefly, cells were adhered to poly-lysine coated coverslips, fixed in 30 mM HEPES (pH=7.2), 3 mM EGTA, 1 mM MgSO_4_, and 25 mM KCl with 4% paraformaldehyde (“PFA”) for 5 minutes, permeabilized with 0.5% NP-40 for 5 minutes, and then fixed in ice cold methanol for 5 minutes. Coverslips were blocked in 5% BSA and 1% Fish (“Block”) gelatin for 30 minutes, then in block with 10% Normal Goat Serum (“NGS", Sigma), and then incubated in β-tubulin (CST, 2146S) diluted in 20% block in PBS overnight at 4°C. Coverslips were washed 3x10 minutes in PBS, incubated in secondary (Alexafluor 488 goat anti-rabbit IgG, A11008, 1:500) for 1 hour, washed 3x10 minutes in PBS, and allowed to dry. Coverslips were mounted in Fluoromount G (Thermo Scientific) before imaging. Cells were imaged on a Nikon Yokogawa SoRa super resolution spinning disk confocal with 100x oil objective (data collection) and 2.8x magnifier (representative images). For quantification, max IPs were generated from z stacks and 100 cells per time point were counted using the Cell Counter tool in FIJI for either having cytoplasmic microtubules polymerized (spanning > half the cell) or not having microtubules polymerized (spanning < half the cell).

#### Acetylated α-Tubulin:

Cells adhered to coverslips were fixed in 4% PFA in 10 mM HEPES for 15 minutes, placed in ice cold 80% acetone for 5 minutes, placed in ice cold 100% acetone for 5 minutes, and then allowed to dry before rehydrating for 5 minutes in PBS. Then cells were blocked in 100% Block for 30 minutes, 10% NGS, and stained for acetylated tubulin (1:1000) overnight at 4°C. Coverslips were washed 3x10 minutes in PBS incubated with secondary (Alexa Fluor 499 goat anti-mouse IgG, Invitrogen A11001) for 1h at RT, covered, and then washed 3x10 minutes in PBS, covered before allowing to dry completely and mount with Fluoromount G. For quantification, max IPs were generated and acetylated microtubules were measured using the segmented line tool in FIJI for 30 cells per timepoint.

#### IFT140/54:

Cells adhered to coverslips were placed in ice cold 100% methanol 2x5 minutes, 50% methanol in PBS for 5 minutes, then PBS for 5 minutes before allowing to dry, mount, and image. For quantification, z stacks were summed together, background subtracted with rolling ball radius = 50, and then equal sized circles drawn over the signal at the base of the cilium. Fluorescent intensity was calculated using the calculation for CTCF as previously described (Dougherty et al., 2023)

#### SDS-PAGE and Immunoblotting

Soluble (S) and insoluble (P) fractions were collected similarly to (Wang et al., 2013). Briefly, treated cells were resuspended in TMMET Buffer with 1 minute of pipetting and 1 minute of gentle vortexing (half-max vortex speed). Cells sat for 10 minutes with 1 gentle round of vortexing before spinning down at 21000xg for 10m, RT. The supernatant was collected (“S” fraction) and then the pellet was washed with buffer. Finally, the remaining pellet was resuspended in TMMET buffer (“P” fraction). Protein from S and P fractions were mixed with DTT and LDS, boiled at 70°C for 10 minutes, and run on a NuPAGE 10% Bis-Tris gels in SDS running. Protein was transferred to PVDF membrane, blocked in 5% milk in PBST, incubated in β-tubulin primary (1:1000, CST 2146S) or actin C4 (1:1000, Sigma MAB1501) overnight at 4°C. Blots were washed 3x10 minutes in PBST, incubated in secondary antibody diluted in 1% milk with 1% BSA (Goat anti-rabbit IgG HRP conjugate, Invitrogen, G21234, 1:5000; Goat anti-mouse IgG HRP conjugate, Invitrogen, 31430, 1:5000) for 1 h at RT. Blots were washed 3x10 minutes in PBST and then incubated with Pico Chemiluminescent substrate (Invitrogen) and imaged on a Syngene G:BOX.

### Statistical Analysis

Data was collected and organized in Excel, and then graphed and analyzed with GraphPad Prism Version 9.5.1 (528). SuperPlots were made and analyzed according to (Lord et al., 2020) where statistics were performed on the averages of 3 trials and individual trials are represented by corresponding symbol colors. For experiments, “n” is the number of cells quantified and “N” is the number of experiments.

## Supplementary Material

Supplement 1

## Abbreviations

CMTs: Cytoplasmic Microtubules
IFT: Intraflagellar Transport
MS: Mechanical Shear
PTX: Paclitaxel
