# Supplementary material for: Determinants of cytoplasmic microtubule reorganization during ciliogenesis in Chlamydomonas reinhardtii
Source: bioRxiv. 2023 May 3:2023.04.07.536038. Originally published 2023 Apr 7. Preprint. [Version 2] doi: 10.1101/2023.04.07.536038 (PMC10104144; doi:10.1101/2023.04.07.536038)
Supplement: Supplementary file 1 [file media-1.pdf]

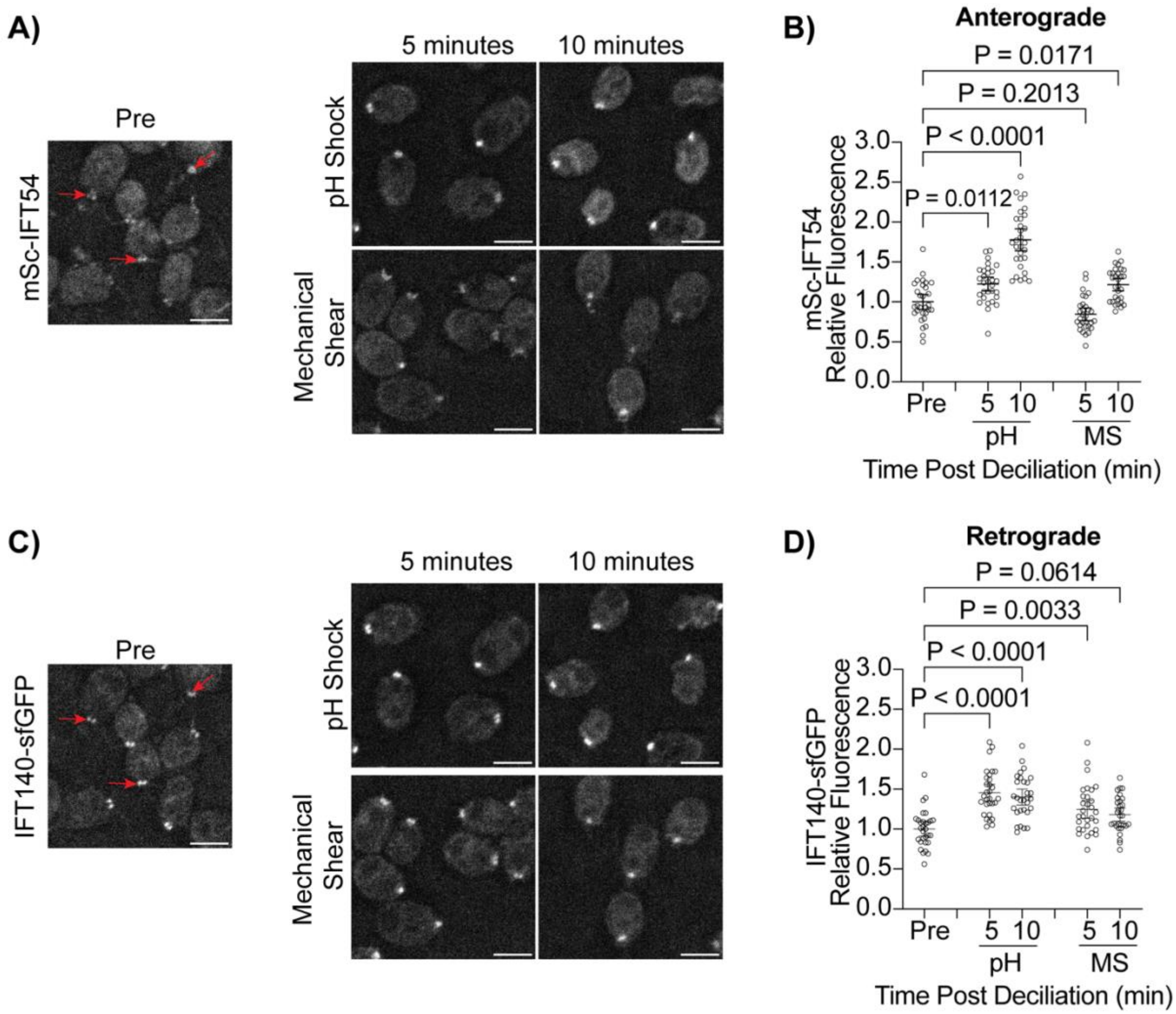

<u>Supplemental Figure 1. PH shock-induced ciliary shedding increases IFT recruitment to the ciliary base</u>. A and C) mSc-IFT54/IFT140-sfGFP were fixed and imaged. Fluorescence at the base of the cilium was quantified (red arrows). Representative images show cells pre deciliation ("Pre"), 5 min, and 10 min post treatment for both pH shock and mechanical shear for both mSc-IFT154 (**A**) and IFT140-GFP (**C**) transport. Scale bars are 5 µm. **B and D)** Quantification of mSc-IFT54 (**B**) or IFT140-sfGFP (**D**) fluorescent intensity at the ciliary base. Error bars are mean with 95% confidence interval (n=30, N=1). Statistics were determined using a One-Way ANOVA and Šídák's multiple comparisons test.

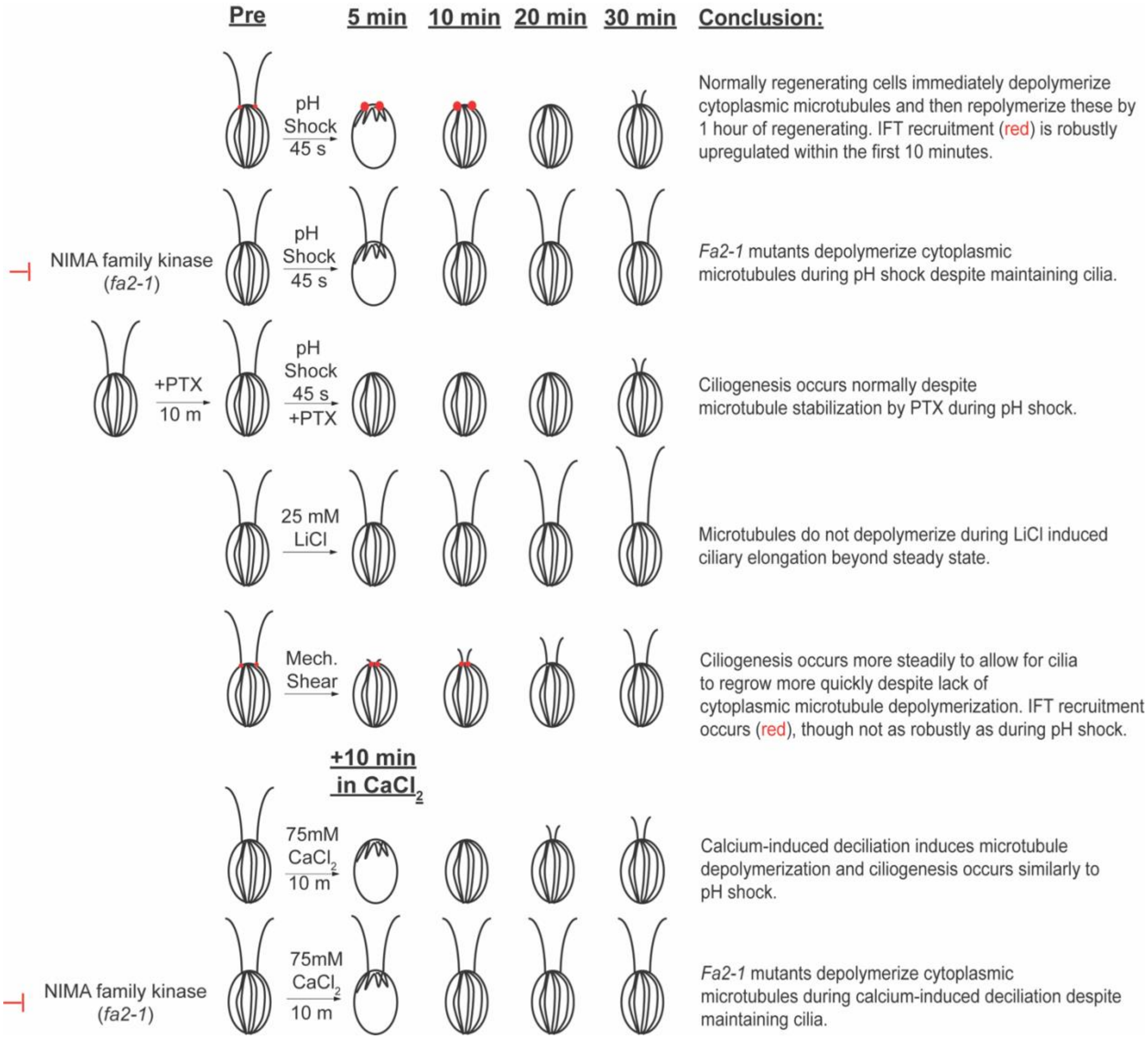

Supplemental Figure 2. Summary of microtubule dynamics in conjunction with ciliogenesis. Results from each trial are summarized for each of the different conditions tested up to 30 min. Red dots represent IFT fluorescence tested for both pH shock (top) and mechanical shear (bottom). Adapted from (Dougherty et al., 2022).
